# Radiographers supporting radiologists in the interpretation of screening mammography: a viable strategy to meet the shortage in the number of radiologists

**DOI:** 10.1186/s12885-015-1399-2

**Published:** 2015-05-16

**Authors:** Gabriela Torres-Mejía, Robert A. Smith, María de la Luz Carranza-Flores, Andy Bogart, Louis Martínez-Matsushita, Diana L. Miglioretti, Karla Kerlikowske, Carolina Ortega-Olvera, Ernesto Montemayor-Varela, Angélica Angeles-Llerenas, Sergio Bautista-Arredondo, Gilberto Sánchez-González, Olga G. Martínez-Montañez, Santos R. Uscanga-Sánchez, Eduardo Lazcano-Ponce, Mauricio Hernández-Ávila

**Affiliations:** 1 Centro de Investigación en Salud Poblacional, Instituto Nacional de Salud Pública, Avenida Universidad No. 655, Colonia Santa María Ahuacatitlán, Cuernavaca, 62100, Morelos Mexico; 2 American Cancer Society, 250 Williams St., Atlanta, GA 30303 USA; 3 Centro de Diagnóstico Digital México-España, Secretaria de Salud Pública del Distrito Federal, Mariano Escobedo No. 148 col. Anáhuac, Ciudad de México D. F., 11320 Mexico; 4 Group Health Research Institute, Group Health Cooperative, 1730 Minor Ave #1600, Seattle, WA 98101 USA; 5 Division of Biostatistics, Department of Public Health Sciences, School of Medicine, University of California, 1 Shields Ave, Davis, CA 95616 USA; 6 Department of Epidemiology and Biostatistics and the General Internal Medicine Section, University of California, 4150 Clement St, San Francisco, CA 94121 USA; 7 Department of Veterans Affairs, University of California, 4150 Clement St, San Francisco, CA 94121 USA; 8 Dirección de Economía de la Salud, Instituto Nacional de Salud Pública, Avenida Universidad No. 655, Colonia Santa María Ahuacatitlán, CP. 62100 Cuernavaca, Morelos Mexico; 9 Hospital de Oncología, Centro Médico Siglo XXI, Instituto Mexicano del Seguro Social, Av. Cuauhtémoc 330, Cuauhtemoc Doctores, Ciudad de México, D.F. 06720 Mexico; 10 Federación Mexicana de Colegios de Ginecología y Obstetricia, Nueva York 38, Col. Nápoles, Benito Juárez, Ciudad de México, D.F. 03810 Mexico

**Keywords:** Radiographers, Film readers, Screening mammography, BI-RADS system

## Abstract

**Background:**

An alternative approach to the traditional model of radiologists interpreting screening mammography is necessary due to the shortage of radiologists to interpret screening mammograms in many countries.

**Methods:**

We evaluated the performance of 15 Mexican radiographers, also known as radiologic technologists, in the interpretation of screening mammography after a 6 months training period in a screening setting. Fifteen radiographers received 6 months standardized training with radiologists in the interpretation of screening mammography using the Breast Imaging Reporting and Data System (BI-RADS) system. A challenging test set of 110 cases developed by the Breast Cancer Surveillance Consortium was used to evaluate their performance. We estimated sensitivity, specificity, false positive rates, likelihood ratio of a positive test (LR+) and the area under the subject-specific Receiver Operating Characteristic (ROC) curve (AUC) for diagnostic accuracy. A mathematical model simulating the consequences in costs and performance of two hypothetical scenarios compared to the *status quo* in which a radiologist reads all screening mammograms was also performed.

**Results:**

Radiographer’s sensitivity was comparable to the sensitivity scores achieved by U.S. radiologists who took the test but their false-positive rate was higher. Median sensitivity was 73.3 % (Interquartile range, IQR: 46.7–86.7 %) and the median false positive rate was 49.5 % (IQR: 34.7–57.9 %). The median LR+ was 1.4 (IQR: 1.3-1.7 %) and the median AUC was 0.6 (IQR: 0.6–0.7). A scenario in which a radiographer reads all mammograms first, and a radiologist reads only those that were difficult for the radiographer, was more cost-effective than a scenario in which either the radiographer or radiologist reads all mammograms.

**Conclusions:**

Given the comparable sensitivity achieved by Mexican radiographers and U.S. radiologists on a test set, screening mammography interpretation by radiographers appears to be a possible adjunct to radiologists in countries with shortages of radiologists. Further studies are required to assess the effectiveness of different training programs in order to obtain acceptable screening accuracy, as well as the best approaches for the use of non-physician readers to interpret screening mammography.

## Background

In Mexico, since 2006, breast cancer is the leading cause of death from cancer in women [1] with epidemiological and demographic transitions contributing to an increasing trend in rates. Approximately 90 % of breast cancer cases are diagnosed at an advanced stage, which in part is due to the lack of an organized screening program, limited access to mammography and shortage of radiologists to interpret screening mammography, particularly in rural areas [2]. These conditions may contribute to delays in diagnosis and less successful treatment [2–5].

The Mexican Official Norm for breast cancer (NOM-041-SSA2-2011) recommends mammography every two years for healthy women ages 40–69 years and provides the guidelines for the breast cancer national program to achieve greater coverage and quality [6]. However, the results of the recent Mexican National Health Survey, showed that only 17.2 % and 29.4 % of women between 40 and 49 years and between 50 and 69 years, had a mammogram within the previous 2 years, respectively [7]. Only 291 radiologists participate in the screening mammography program in Mexico, among whom 260 focus exclusively on breast imaging. With these current infrastructure and human resources, it will clearly not be possible to increase the coverage up to 70 % as suggested by the World Health Organization (WHO) [8], given that there are close to 14 million women eligible for screening [2, 3, 9].

Since the mid-1980s, there has been a growing literature suggesting that shortages of radiologists could be overcome, and costs reduced if radiographers (also known as radiologic technologists) [10, 11], or mid-level practitioners (physician assistants or nurse practitioners) could interpret mammograms and serve as first readers, determining the presence or absence of abnormal images in cases that warrant further evaluation by a radiologist. [12] In 1995 in the United Kingdom (UK), there was both a shortage and falling recruitment rates of radiologists, and therefore the possibility of radiographers as readers was explored in order to maintain double reading [13]. Since then, several studies have demonstrated radiographers’ ability to identify abnormalities on screening mammograms [14] albeit with higher false positive rates, but similar sensitivity compared with radiologists [15–19]. Furthermore, trained radiographers have been shown to perform well as pre-readers in a clinical setting [20].

To our knowledge there are no studies examining the potential for radiographers or physician extenders to contribute to mammography interpretation in Latin America. Given Mexico’s public health policy goals for breast cancer screening and the shortage of radiologists to interpret mammograms, we designed a study to evaluate the performance of 15 radiographers in the interpretation of mammograms after a 6 months training period in a screening setting. Additionally, using the results of the study, we modeled the effects on costs and performance of two hypothetical screening scenarios; both were compared with the *status quo* in which the standard practice is a single reading by a radiologist. We conducted this study to determine the feasibility of having radiographers as first readers, in order to consider a strategy for integrating non-radiologist readers into mammography screening in countries that, face the problem of shortages of radiologists with specialization and interest in mammography.

## Methods

### Study population

Fifteen radiographers from 12 states in Mexico were invited to participate in the study. Inclusion criteria were: 1) having a formal role in the mammography facility operating under the auspices of the Secretariat of Health, 2) having completed radiographers training and obtained a degree, 3) at least 6 months experience in x-ray and breast imaging, and 4) permission from the institution where he or she worked in order to engage in the study.

### Screening setting

Health-care coverage in Mexico is delivered by a range of different institutions. Although there are isolated efforts on the implementation of an organized population-based screening program, the breast cancer screening program in Mexico is based on an opportunistic model [21]. For this study, the training was held in Mexico City in a Digital Diagnostic Center, which forms part of a network of 20 digital mammography machines in health facilities distributed across Mexico City with a goal of performing and interpreting 90, 000 mammograms annually [22]. All participants received a grant for maintenance and transportation. The setting offered a class-room, a reading area with a work station and trainees.

### Training program

The training program was designed by a group of radiologists and epidemiologists. It had a total duration of 6 months and consisted of clinical lectures, training in-service by three radiologists who guided the radiographers. The participants read a progressive number of digital mammographic studies using the Breast Imaging Reporting and Data System (BI-RADS) system. During the first month, each student interpreted at least 10 screening mammograms a day; as they improved their performance, the number of mammograms assigned daily increased to approximately 40 mammograms per day. Each day, the radiographers, in teams of two, reviewed 10–30 mammograms with the advice of a radiologist, and on a weekly basis they received feedback, in class, using a subsample of images. Prior to the final test set evaluation, the median number of mammograms interpreted by the radiographers during the six-month training program was 777. The classroom component consisted of 122 training hours on breast anatomy and mammographic features of normal, benign and malignant breast conditions based on updated literature on the standards for training specialized health professionals dealing with breast cancer [23]. They also received an introduction to breast cancer epidemiology and ethics.

### Evaluation

At the end of training (6 months), a formal evaluation was performed using a self-administered test set of mammograms developed by the Breast Cancer Surveillance Consortium (BCSC) [24] for the evaluation of U.S. radiologists in a prospective study of skills assessment and training. The evaluation was carried out in the computer lab of the National Institute of Public Health using software developed by the American College of Radiology for the BCSC.

The test set consisted of 110 screening examinations of which 15 cases were biopsy confirmed cancers, 14 were non-cancers that 3 U.S. expert radiologists judged should be recalled, and 81 were non-cancers that the experts judged had no findings to justify recall. Of the 15 confirmed breast cancer cases, the 3 U.S. expert radiologists who helped assemble the test set judged 3 to be obvious, 7 intermediate, and 5 subtle, from which 100 %, 85.7 %, and 80 % were recalled in the U.S. clinical practices, respectively. U.S. experts’ consensus was that all cancers should have been recalled. Among the 14 non-cancer cases which were recalled by the expert panel, 12 (85.7 %) were recalled in clinical practice (personal communication from Andy Bogart, BCSC Statistical Coordinating Center). In the main analysis, the biopsy confirmed cancer cases were treated as the true positives for evaluation purposes. Each of the 110 cases had 4 images (mediolateral oblique and cephalocaudal views for each breast). Participants also had access to screening mammograms performed within 2 years before the test films were taken in order to have a baseline comparison.

Interpretive performance was measured in terms of sensitivity, specificity, false positives, likelihood ratio of a positive test (LR+) and the area under the subject-specific Receiver Operating Characteristic (ROC) curve (AUC). The assessments performed by the 3 U.S. expert radiologists were defined as the gold standard. The three breast imaging experts were selected based on their expertise in breast imaging. Two of the three breast imaging experts were males and all were over 55 years. All worked in an academic setting and had over 30 years’ experience interpreting mammograms. Their annual volume of mammography interpretation ranged from 2500 to 7000 per year. All experts were fellows of the Society of Breast Imaging (SBI), fellows of the American College of Radiology, past presidents of National Breast Societies, and gold medalists of the SBI. For each case the radiographer was asked to identify the BI-RADS interpretation category corresponding to the observed images: BI-RADS 0 (needs further evaluation), 4 (suspicious) or 5 (highly suggestive of malignancy) or BI-RADS 1 (negative) or 2 (benign finding). BI-RADS 3 category was not an interpretation category, because it should not be used in screening [25]. For evaluation purposes, sensitivity was estimated as the percent of cancer cases that were recalled by radiographers out of the total number of cancer films in the test set (n = 15), and false negatives as its complement. Specificity was calculated as the percent of non-cancer films which were correctly not recalled by the radiographers of the total of non-cancer films in the test set (n = 95) and false positives as its complement.

For recalled mammograms, the most significant finding type was described as either a mass, calcifications, asymmetry, or architectural distortion. After the evaluation, the results were sent via the Internet to a server in the BCSC Statistical Coordinating Center, Group Health Cooperative, Seattle WA, U.S. to be analyzed by one of the BCSC members. The database was sent back via secure file transfer protocol to Mexico for further analysis. Subsequently, specific results were sent back to each participant with confidentiality maintained.

### Statistical analysis

An exploratory analysis was performed to describe the socio-demographic, academic and experience characteristics of the participants and of the variables related to diagnostic interpretive performance, measured by sensitivity, specificity, false positive rate, the AUC and the likelihood ratio of a positive test (LR+). We report the median and the inter-quartile range (IQR) of performance measures across radiographers. For continuous variables we used measures of central tendency and dispersion. For categorical variables we used measures of frequency.

To evaluate performance, sensitivity, specificity and false positive (FP) rate were calculated. Overall observer performance was measured by calculating the AUC and the LR+ for each participant. The AUC was estimated as the median of the sensitivity and specificity as described by Cantor and Kattan [26]. The LR+ for each radiographer was calculated by dividing the sensitivity by 1-specificity. This measure combines sensitivity and specificity into a single index that measures how many times it is more likely that a patient recalled by experts has an abnormal mammogram compared to women whom the experts did not recall [27].

### Mathematical modeling

**Fig. 1:**
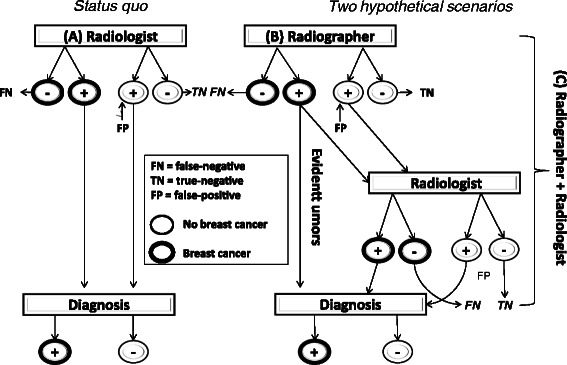
Decision tree model to assess the costs and effects of screening mammography interpretation by the radiographers in this study in terms of true positives, false negatives and false positives. The model compares three hypothetical scenarios: (**A**) the status quo in which one radiologist reads all mammograms; (**B**) a radiographer reads all mammograms; and (**C**) a radiographer reads all mammograms first, sends obvious abnormal findings for diagnostic evaluation and leaves to the radiologist, for a second reading, only those images which he/she considers difficult to interpret. Both scenarios (**B** and **C**) were compared with the status quo, where a radiologist reads all mammograms (**A**)

We designed a mathematical model to assess the costs and outcomes of screening mammography interpretation by the radiographers in this study in terms of true positives, false negatives and false positives. The model compares three hypothetical scenarios: (A) the *status quo* in which a radiologist reads all mammograms; (B) a radiographer reads all mammograms; and (C) a radiographer reads all mammograms first, recommends obvious abnormal findings for diagnostic evaluation, and refers to a radiologist for a second reading any images that appear abnormal, but for which the need for recall is uncertain (i.e., radiographer’s percentage of “FP, but not obvious breast cancer” images). Both scenarios (B and C) were compared with the *status quo,* where a radiologist reads all mammograms (A) (Fig. 1).

We compared the results in terms of diagnostic accuracy and costs. The parameters of the model were: salary per month for a full time radiographer and a radiologist [28], mammogram and diagnosis costs [29], prevalence of breast cancer in a screening setting [30], average number of mammograms read per month (the number of mammograms per month was assumed to be the same for both radiologists and radiographers) [31], and test set sensitivity and specificity obtained from this study for radiographers and a previous clinical setting for radiologists [31] (Table 1). All model input parameters were introduced as triangular probability distributions, whose minimum, mean and maximum values are indicated in Table 1. The triangular distribution allowed us to assign probability distributions to the costs and effectiveness parameters rather than simple point estimates. This is a common approach when the actual distribution of the parameter is unknown, but three parameters (minimum, maximum and some modal value) are known or can be guessed.

**Table 1:** Parameters of the mathematical model, Mexico 2012

Parameter	Mean/Median value	95 % CI	Reference
Radiologists' salary per month^a^	663	600-720	[24]
Radiographers' salary per month^a^	407	360-440	
Mammogram's cost^a^	24	22-26	[25]
Confirmation (diagnosis) cost^a^	802	720-880	
Number of invasive cancers detected per 1000 screens	5	0.45-0.55	[26]
Average number of mammographies read per month	93	58-200	[27]
Radiologists' sensitivity	0.729	0.604-0.833	
Radiologists' specificity	0.844	0.781-0.906	
Radiographers' sensitivity	0.733	0.467-0.867	Present study
Radiographers' specificity	0.505	0.421-0.653	

^a^ Salaries and costs are indicated in USD

Results of both scenarios were calculated using second-order Monte Carlo simulations. The costs and effectiveness parameters were drawn from the triangular probabilistic distributions described above. This method implies that during the simulation these parameters are sampled randomly from the triangular distributions, and each sample drawn represents one radiologist or radiographer performance and costs. In total 1000 radiologists and 1000 radiographers were simulated and the results of all simulations were averaged. The results therefore, explicitly consider the uncertainty in the input parameters of the model. Instead of performing simple point-estimate sensitivity analysis, the results are reported with confidence intervals. This approach is referred to as probabilistic-sensitivity, or multiway sensitivity analysis [32]. The labor costs used in the model were in terms of one month full time equivalent unit of either radiologist or radiographer.

### Ethics

The study was approved by the Institutional Review Board at the National Institute of Public Health. All participants gave their consent to participate in the study. The study was in agreement with the regulations established by the Breast Cancer Surveillance Consortium Assessing and Improving Mammography (BCSC-AIM) Collaborative Research Agreement supported by the American Cancer Society and the U.S. National Cancer Institute.

## Results

### Performance of radiographers

Eighty percent of the radiographers who participated in the study were women (80 %) and the median age was 38 years (IQR: 28–47 years) (Table 2). The median duration of educational training to become a radiographer was 2.5 years (IQR: 2–3 years) and the median time of experience performing mammography studies was 8 years (IQR: 2–18). Prior to the educational training, they had no previous experience in interpreting mammograms. Most radiographers worked for second level of attention general hospitals (42.9 %) and very few reported having previously attended breast disease courses (Table 2).

**Table 2:** Characteristics of radiographers (n = 15), Mexico 2012

Age (years)	
Median	38
Interquartile range	(28–47)
Range	(24–54)
Sex	
Female	12 (80 %)
Male	3 (20 %)
Years since graduation	
Median	8
Interquartile range	(5, 19)
Range	(2, 26)
Technical education cumulative grade score^a^	
Median	8.5
Interquartile range	(8–9.7)
Range	(7.8–10)
Technical education length (years)	
Median	2.5
Interquartile range	(2–3)
Range	(1–4)
Experience in performing mammography studies (years)^b^	
Median	8
Interquartile range	(2–18)
Range	(0–25)
Number of mammograms per week performed before training^c^	
Median	100
Interquartile range	(50–125)
Range	(10–200)
Health care level	
First	4 (28.6 %)
Second	6 (42.9 %)
Third	4 (28.5 %)
Number of additional breast courses	
Median	1
Interquartile range	(0–5)
Range	(0–10)

^a^The cumulative grade score is on a scale of 1–10

^b^Radiographers were not necessarily devoted exclusively to this activity

^c^Radiographers in Mexico do not interpret, they only perform mammograms

**Fig. 2:**
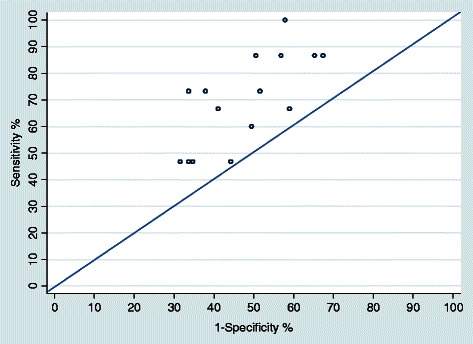
Sensitivity vs. percentage of false positives (1-specificity) on the test set performance evaluation among 15 radiographers after 6 months of training. Mexico 2012

The median sensitivity was 73.3 % (IQR: 46.7-86.7 %) whereas the average false positive rate was 49.5 % (IQR: 34.7–57.9 %). (Table 3, Fig. 2). The PPV was 18.3 % (IQR: 16.9 %–21.3 %) and the NPV was 92 % (IQR: 88.7–94.3 %). The median likelihood ratio of a positive test was 1.4 (IQR: 1.3–1.7 %) and the median AUC was 0.6 (IQR: 0.6–0.7). The median time to interpret a study per radiographer was 115.9 s (IQR: 105.2–131.6 s) (Table 3).

**Table 3:** Radiographers’ test set performance evaluation after 6 months of training, Mexico 2012

Performance post-training	Median	Interquartile range
Sensitivity ( %)^a^	73.3	46.7–86.7
Specificity ( %)^a^	50.5	42.1–65.3
False positivies (1 – specificity) ( %)	49.5	34.7–57.9
Appropiate recalls ( %)^b^	78.6	78.6–92.9
In appropiate recalls ( %)^c^	36.8	25.3–44.2
Positive predictive value ( %)	18.3	16.9–21.3
Negative predictive value ( %)	92.0	88.7–94.3
LR + ^d^	1.4	1.3–1.7
AUC^e^	0.6	0.6–0.7
Time spent per interpretation^f^	115.9	105.2–131.6

^a^ The biopsy confirmed cancer cases were treated as true positives for evaluation purposes

^b^Percent non-cancer appropriate recalls

^c^Percent non-appropriate recalls

^d^LR+ Likelihood ratio of a positive test = (sensitivity)/(1-specificity)

^e^AUC Area under the subject-specific receiver operator characteristic (ROC) curve

^f^Time in seconds

In relation to the characteristics of the breast cancers, sensitivity was highest for identification of masses and architectural distortions (100 % for both) and lowest for asymmetries (25 %) (Table 4). Radiographer’s sensitivity decreased with increasing difficulty of the lesion, with median sensitivity of 100 % for obvious lesions, 71.4 % for intermediate lesions, and 60 % for subtle lesions (Table 4).

**Table 4:** Radiographers' sensitivity by lesion type and difficulty after 6 months of training, Mexico 2012

Performance post-training	Median	Interquartile range
	% Sensitivity^a^	
Breast cancer lesions types		
Mass	100.0	66.7–100.0
Calcification	83.3	66.7–100.0
Asymmetry	25.0	25.0–50.0
Architectural distortion	100.0	50.0–100.0
Difficulty of cancer lesion identified by radiographers		
Obvious	100.0	66.7–100.0
Intermediate	71.4	57.1–85.7
Subtle	60.0	40.0–80.0

^a^Percentage of histologically confirmed breast cancer lesions that were recalled by radiographers, by type of lesion (mass = 3, calcifications = 6, asymmetry = 4, architectural distortion = 2) and difficulty (obvious = 3, intermediate = 7, subtle = 5)

### Model outcomes

1. Radiographer vs radiologist

When comparing the monthly cost of mammography screening interpretation by radiographer vs radiologist, it was more efficient to employ a radiologist, despite the differential in salaries. The total monthly cost of Scenario A, where the radiologist interprets all mammograms, was less expensive than the total monthly cost of Scenario B, where the radiographer interprets the same number of mammograms (US$17,019 vs US$44,165, respectively) (Table 5). These scenarios were comparable in terms of percentage of true positives (0.36 % vs 0.34 %, respectively), in terms of false-negative results (0.14 % vs 0.16 %, respectively) and in terms of total mammograms interpreted per month. However, the results in terms of false positive readings were very different (15.6 % vs 47.1 %, respectively) because the false-positives were based on a clinical setting for the radiologists and a test set for the radiographers where the test set is enriched with abnormal examinations [31]. As a result of this difference, the cost per breast cancer detected was significantly higher in scenario B compared with scenario A (US$139,263 vs US$51,403, respectively).

**Table 5:** Model outcomes for different scenarios, Mexico 2012

Strategy		Total cost per month		% of true-positives results	% of false-negatives results	% of false-positives results	Average cost per case found
		(in US Dollars)					(in US Dollars)
(A)	Radiologist only	Mean	17,019	0.356	0.14	15.55	51,403
		95 % CI	16,376–17,651	0.23–0.48	0.23–0.23	14.88–16.26	38,124–79,563
(B)	Radiographers only	Mean	44,165	0.341	0.155	47.13	139,263
		95 % CI	43,556–45,067	0.22–0.45	0.08–0.23	46.11–48.1	105,531–215,858
(C)	Radiographers and radiologist	Mean	16,331	0.342	0.154	16.99	51,347
		95 % CI	15,755–16,921	0.23–0.47	0.08–0.24	14.33–17.68	37,363–76,350
Incremental (B-A)		Mean	27,146	−0.015	0.015	31.58	87,860
		95 % CI	25,376–28,916	−0.364–0.334	−0.219–0.249	29.14–34.02	15,356–160,365
Incremental (C-A)		Mean	−687	−0.014	0.015	1.44	−56
		95 % CI	−2415–1040	−0.351–0.323	−0.219–0.249	−4.04–6.93	−3625–3512

Note: Average cost per case found = total cost per month/(-% of true-positives results/100*average number of mammographies read per month)

The denominator of percentages of true positives, false negatives and false positives is the whole sample. The exchanged rate used was 13 Mexican Pesos per USD (January, 2014)

2. Radiographer and radiologist working together vs radiologist only

Our model showed that the monthly cost and average cost per breast cancer case detected for scenario C (radiologist & radiographer) was slightly lower (US$16,331 and US$51,347, respectively) than for Scenario A (radiologist only) (US$17,019 and US$51,403, respectively).

## Discussion

Our study used a test set to evaluate the effect of a 6-month screening mammography interpretation training program for radiographers. The median sensitivity was 73.3 %, but was achieved at the expense of a high percentage of false positives (49.5 %) on a test set enriched with abnormal examinations with an expert recall rate of 26.4 % (29/110). Our results are consistent with other studies evaluating performance on consecutive series of patients or test set studies where sensitivity rates have ranged from 73 % to 90 % [33]. Recall rates tend to be higher in test set situations than one expects to find in screening conditions [34]. In addition, this test set was developed to be challenging, especially for the non-cancer cases. For example, the clinical sensitivity obtained from U.S. radiologists on the same films read in clinical practice, was 86.7 % (13/15) while the false positives rate for the test set films was 38.9 % (37/95) (personal communication: Andy Bogart BCSC Statistical Coordinating Center).

Among the advantages of our study was that training took place in a center far from the radiographers’ work environment, enabling them to dedicate themselves exclusively to learning to interpret mammograms. The test was conducted in a computer lab and we ensured that radiographers did not communicate with each other during the exam. The interpretations were sent directly to an external evaluator (BCSC) so that neither the researchers nor the radiographers knew the results of the assessment at the time of examination. In our study, we also measured sensitivity by treating recalled biopsy-proven benign cases as true positives when the abnormality was judged by expert radiologists to warrant recall (median = 75.9; IQR: 65.5–86.2; data not shown). This is a reasonable and clinically relevant approach for both radiologists and radiographers, since some screening exams, although eventually determined to be benign, must be recalled due to the suspicious nature of the abnormality. Since non-radiologist readers may be expected to interpret exams with a lower threshold for suspicion compared with radiologists, this approach may be even more appropriate for non-radiologists [15, 35]. Finally, not knowing the number/proportion of cancers in the test set, or the fraction of non-cancers for which recall was expected, prevented the examination of cases with a “counting down” approach to the identification of abnormal exams.

Our investigation has some limitations. In many centers in Mexico, mammography examinations are performed using full-field digital mammography units and the radiographers were trained utilizing digital mammography images, while the test set was constructed with digitized analog images, which may have affected the radiographers’ performance. Radiologists who trained the radiographers may have varied in their ability to accurately interpret screening mammograms, such as it has been previously observed [31, 36, 37]. The participants in this study achieved the stated performance levels with relatively low levels of overall training and experience in the field, compared with some other settings in which radiographers read mammograms. For example, in the U.K., radiographers are all initially trained to a bachelor’s degree standard, and to work in breast screening they must undertake a master’s level course of approximately one year’s duration including the reading of 1500 to 2000 mammograms with feedback [38]. In contrast, an average Mexican radiographer studies 2–3 years after junior high-school, and rarely is exposed to curricula specifically related to breast cancer screening (e.g., only 3 out of 27 schools analyzed), which they typically will receive as on-the-job training once they begin working. Further, in the present study the radiographer read fewer mammograms (mean = 770; SD 174), and only received feedback in class on a subsample of the homework.

The radiologists’ specificity used for the mathematical model is not directly comparable with the radiographers’ specificity obtained in our study, given that the conditions from which the measures were derived were different. If the evaluation had been comparable, i.e., U.S. test set, radiologists likely would have a higher false positive rate, and would be more expensive (Scenario A, Radiologist would cost US $19, 061 total cost per month assuming a mean value of sensitivity = 73.3 and specificity = 53.7, data not shown vs. US $17, 019 (Table 5)). An important difference between scenarios A and C, the implications of which are not quantified in our results, is that scenario C could increase access to mammography screening by increasing the number of film readers, reducing the screening load of the radiologists, and providing greater time for the radiologist to devote to evaluating difficult and abnormal screening exams. In a setting where there are too few radiologists to achieve recommended screening goals, scenario C offers a potential solution. The model presented in this study does not provide sufficient evidence for the alternative scenarios, but provides a first estimate of how these scenarios would compare to usual practice. Further, we would expect that radiographers’ accuracy, both sensitivity and specificity, would improve with continuing experience and training, thus steadily improving the cost-effectiveness. Lastly, the accuracy of mammography interpretation by Mexican radiologists measured in an earlier study was based on films interpreted with a film viewer rather than digitized images using a computer screen as was used in our study, and no information is available about the breast cancer lesion types and difficulty in that earlier evaluation used for the Mexican radiologist’s evaluation [31]. Finally, we had no baseline measure of performance for the radiographers and no comparison group, so we are not able to directly measure the effect of the training program. However, radiographers in Mexico do not have a formal role in the interpretation of mammograms, and given that was their first experience, we believe our results are a reasonable proxy of the effect of training.

Radiographers are good non-radiologist candidates for the interpretation of mammograms because of their considerable experience with breast imaging, professional dedication [18], and because they work under the supervision of a radiologist. Sumkin et al. showed that even without undergoing additional training, technologists classified screening mammograms at a reasonable level of accuracy [19]. In addition, when radiographers participate in the interpretation of mammograms it contributes to increased realization of the importance of producing high quality mammographic images [18], and their satisfaction at work [35]. Besides radiographers, other health professionals such as physician assistants, nurse practitioners, and general practitioners are worthy of consideration as candidates where there are shortages of radiologists, provided that they have adequate initial training and supervision, on-going training and evaluation, and can perform at pre-set target levels determined for the program. There also is evidence that radiologists [39, 40], and other specialists [41, 42], will accept other health professionals performing services that traditionally only have been performed by them if there has been formal training, and there are access problems, such as shortages of specialists in rural areas [43, 44].

Compared with radiologists, radiographers or physician assistants have achieved similar sensitivity after initial training, although generally with higher false positive rates in screening settings and on test sets [33]. A test-set likely still underestimates clinical specificity performance because the participants would have known that a recall decision in the test would not carry a cost (e.g., unnecessary procedures and psychological effects) for a real woman [45]. Investigators have noted that it is realistic to anticipate that specificity would improve with additional training and experience to the equivalent of radiologists reading screening mammograms. [12, 16, 46, 47] Evidence from mature programs that have included radiographers in the interpretation of mammograms, such as the U.K. National Health Services Breast Screening Program (NHSBSP), confirms that both sensitivity and specificity are similar among radiographers and radiologists [48, 49]. Improvement in accuracy also has been observed in the learning curves of radiologists involved in breast imaging [50].

Investigations focused on the ability of radiographers and other non-radiologists to interpret mammograms typically have taken place in settings where there was not an acute shortage of radiologists [33, 51], although consideration of the potential for non-radiologists to play a role in the interpretation of mammograms usually has been motivated by affordability, anticipated personnel shortages, and the pressure of a growing number of women invited to screening due to demographic change and program expansion. In the U.K., for example, increasing workloads led to interest in training radiographers to reduce the time demands on radiologists while maintaining the programmatic commitment to double reading. Presently radiographers contribute to a significant fraction of screening interpretations in the NHSBSP, and the evidence indicates that there are no significant differences in the interpretative accuracy of radiographers and radiologists [48, 49].

While pre-reading by radiographers has been proposed as an alternative to the interpretation of mammograms solely by radiologists in a screening setting [17] it has not been supported by others [12] due, in part, to the risk of missing lesions [33]. However, it has to be acknowledged that radiologists also do not achieve perfect sensitivity in practice. Some false negatives are not visible in retrospect, and even the most skilled radiologist does not detect all breast cancers. To consider the potential for radiographers as first readers, they must be able to achieve similar, not necessarily superior, screening sensitivity in detecting cancers compared with radiologists, and the evidence consistently supports that with adequate training they do achieve that benchmark. Indeed, in some U.K. practices, radiographers are paired for double reading, and radiologists only interpret non-concordant exams [51].

While the ability to achieve the same sensitivity as radiologists is important, there are numerous options to achieve that goal. After training, a radiographer could be paired with a radiologist or experienced radiographer in a program of double reading and periodic proficiency testing with enriched tests sets until program leaders were satisfied that the radiographer’s performance was reliable. To assure confidence in their performance, periodic proficiency testing could be required for a period of time after completion of training, and regular medical audits afterwards. A program could follow the U.K. model and have all exams double read by radiographers, with discordant interpretations referred by a radiologist. Alternatively, a program could accept lower specificity as a way to reach the goal of high sensitivity. Radiographers also could be entirely or initially limited to reading mammograms only from women without significant breast density, leaving more difficult cases for radiologists. Each of these options reduces the amount of radiologist time in the interpretation of screening exams, for which the large majority will be normal, while assuring equivalent accuracy. A skills-mix model such as this allows the physician to focus their time, which is scarce, on supervision, refereeing discordant cases, and diagnostic evaluation of abnormal test results and women who present with symptoms. Still, while scientific evidence and the U.K. experience leaves little doubt that radiographers can perform effectively as interpreters of screening mammograms, the process of their integration into a screening program requires adherence to high standards, and careful implementation in order to assure the confidence of policy makers, radiologists, and the public.

Combining the expertise and skills of both a radiographer and a radiologist in the interpretation of screening mammograms could be an efficient alternative to the traditional model where radiologists are responsible for all screening and diagnostic mammography, especially in a setting where there is a shortage of radiologists or where growing imaging needs will eventually exceed available specialty resources. Although the model does not provide sufficient evidence for other alternative scenarios, our results suggest that taking advantage of the high sensitivity of interpretations by radiographers and high specificity of radiologists could result in an efficient strategy for screening mammography. This would imply a different use of radiologists’ time and a more rapid delivery of positive results to patients by letting the radiographers taking care of obvious interpretations, and triage those that warrant evaluation by the radiologist. Improved training of radiographers and practice could improve these results so radiographers would not likely be generating additional procedures beyond what the radiologists would generate if they were reading as single readers.

While scientific evidence and the U.K. experience leaves little doubt that radiographers can perform effectively as interpreters of screening mammograms, the implementation of a mixed skills program faces numerous challenges. Costs must be considered in the design of training programs, including the potential for enhanced salaries. There also is the requirement for implementation of regulations regarding the additional radiographer’s responsibility to undertake mammographic image interpretation. In this study, the mathematical model to assess the costs and outcomes of screening mammography interpretation, by radiologists and radiographers, was based on cases in which abnormalities were detected. Going further, it would be desirable to perform a cost-effectiveness study to estimate the cost of breast cancer screening under different scenarios of personnel involved in interpretation with an emphasis on deaths averted from breast cancer or life years saved, which is the ultimate goal of screening. Where shortages of radiologists exist, there is a need to determine whether there is an adequate pool of qualified radiographers, and whether recruiting them to be readers would create personnel shortages of radiographers. There likely would be a need to determine the training needs and costs, and compare the performance of non-radiographers as interpreters. There also is the need to determine how many non-radiologists are needed, and the volume of examinations they would be expected to interpret. Above all else, the process of their integration into a screening program requires adherence to high standards, and careful implementation in order to assure the confidence of policy makers, radiologists, and the public.

## Conclusions

Our findings and those of others have shown that well trained radiographers could serve as first readers under the supervision of a radiologist if there is dedication and formal training. Mammography as part of an organized screening program has been shown to reduce mortality from breast cancer [52, 53]. In many middle and low resource countries the infrastructure and personnel are insufficient to provide mammograms to all eligible women through an organized screening program; thus, it is necessary to find innovative options to solve this problem. The existing evidence suggests that the use of non-radiologist readers could provide the opportunity to offer mammography to a greater number of women. The intention of this study was, in part, to present this as an alternative means to interpret mammography, principally because in Mexico and in many other countries, the number of radiologists is insufficient to meet the current and growing need. With little realistic prospect of increasing the numbers of radiologists prepared to read a high volume of mammography, consideration of non-radiologist readers must be examined seriously as part of a set of measures.

## Abbreviations

BI-RADS: Breast Imaging Reporting and Data System
LR+: Likelihood ratio of a positive test
ROC: Receiver Operating Characteristic
AUC: Area under the subject-specific ROC curve
U.S.: United States
IQR: Inter-quartile range
NOM-041-SSA2-2011: The Mexican Official Norm for breast cancer
WHO: World Health Organization
UK: United Kingdom
BCSC: The Breast Cancer Surveillance Consortium
SBI: The Society of Breast Imaging
FP: False positive
BCSC-AIM: Breast Cancer Surveillance Consortium Assessing and Improving Mammography
NHSBSP: National Health Service Breast Screening Program
